# Prospective cohort studies of birth weight and risk of obesity, diabetes, and hypertension in adulthood among the Chinese population

**DOI:** 10.1111/1753-0407.12800

**Published:** 2018-08-21

**Authors:** Qinghua Xia, Hui Cai, Yong‐Bing Xiang, Peng Zhou, Honglan Li, Gong Yang, Yu Jiang, Xiao‐Ou Shu, Wei Zheng, Wang‐Hong Xu

**Affiliations:** ^1^ Center for Disease Control and Prevention of Changning District Shanghai China; ^2^ Division of Epidemiology, Vanderbilt Epidemiology Center, Department of Medicine Vanderbilt University School of Medicine Nashville Tennessee USA; ^3^ State Key Laboratory of Oncogene and Related Genes and Department of Epidemiology Shanghai Cancer Institute, Renji Hospital, Shanghai Jiaotong University School of Medicine Shanghai China; ^4^ Department of Epidemiology, School of Public Health Fudan University Shanghai China

**Keywords:** birth weight, hypertension, obesity, type 2 diabetes

## Abstract

**Background:**

Low birth weight (LBW) has been associated with subsequent risks of obesity and certain chronic diseases, but evidence for the associations is limited for the Chinese population.

**Methods:**

In this study we analyzed data from two population‐based prospective cohort studies, the Shanghai Women's Health Study and the Shanghai Men's Health Study, to examine the associations between LBW and the risk of obesity and chronic diseases. Birth weight was self‐reported at baseline; anthropometric measurements were made at study enrollment. Type 2 diabetes mellitus (T2DM) diagnoses were self‐reported, whereas hypertension diagnoses were based on self‐report and blood pressure measurements at baseline and follow‐up surveys.

**Results:**

Birth weight was available for 11 515 men and 13 569 women. Non‐linear associations were observed for birth weight with baseline body mass index (BMI), waist circumference (WC), waist:  hip ratio (WHR), and waist:  height ratio (WHtR; *P* < 0.05 for non‐linearity), and LBW was linked with lower BMI, smaller WC, and larger WHR and WHtR. An excess risk of T2DM was observed for LBW (<2500 g) versus birth weight 2500–3499 g since baseline (hazard ratio [HR] 1.17; 95% confidence interval [CI] 0.92–1.49) and since birth (HR 1.29; 95% CI 1.07–1.54), whereas the HRs for hypertension since baseline and birth were 1.13 (95% CI 1.01–1.27) and 1.20 (95% CI 1.11–1.30), respectively. The risk of the diseases decreased as birth weight increased up to ~4000 g; further increases in birth weight did not convey additional benefits.

**Conclusion:**

The results suggest that LBW, an index of poor intrauterine nutrition, may affect health risks later in life in the Chinese population.

## Abstract 2

**Highlights**

- Low birth weight was linked with lower body mass index, a smaller waist circumference, and larger waist:  hip and waist:  height ratios in the Chinese population.
- The non‐linear pattern of associations between birth weight and body size in adulthood in this population, as well as the higher risks of type 2 diabetes mellitus and hypertension in men and women with low birth weight, suggest that nutrition in early life is of considerable importance to health in later life.

## Abstract (zh)

**摘要:**

**背景**

低出生体重(Low birth weight,LBW)与成年期肥胖及某些慢性病的风险相关联,  但来源于中国人群的证据有限。

**方法**

本研究分析了来自两个以人群为基础的前瞻性队列研究——上海妇女健康研究和上海男子健康研究——的数据,  以检验LBW与肥胖及慢性病风险之间的关联。基线调查时研究对象自报出生体重及2型糖尿病(T2DM)的患病状况,  并接受了身体测量。高血压的诊断则基于自我报告的患病状况以及基线和随访调查时测量的血压值。

**结果**

队列成员中共11515名男性和13569名女性具有出生体重信息。出生体重与基线时体重指数(BMI)、腰围(WC)、腰臀比(hip ratio,WHR)和腰高比(waist: height ratio,WHtR)呈非线性关联(非线性检验*P* < 0.05)。低出生体重与较低的BMI、较小的WC及较高的WHR和WHtR有关。与出生体重为2500‐3499 g的研究对象相比,LBW者(出生体重< 2500 g)基线调查之后以及自出生起患T2DM和高血压的风险均显著升高,T2DM的风险比[HR]和95%置信区间[CI]分别为1.17(0.92‐1.49)和1.29(1.07‐1.54);高血压的HR(95%CI)分别为1.13(1.01‐1.27)和1.20(1.11‐1.30)。随着出生体重增加,T2DM及高血压的发病风险降低,  但达到4000 g后,  出生体重的进一步增加未见额外收益。

**结论**

LBW作为反映宫内营养不良的指标,  可能影响中国人群成年期健康风险。

## Introduction

Epidemiologic studies conducted in both developed and developing countries, including Scandinavia,^1^, ^2^ the US,^3^ India,^4^ and China,^5^, ^6^, ^7^ have shown that poor nutrition early in life, such as can be caused by fetal exposure to famine, leads to long‐term negative health consequences, particularly in the presence of overnutrition in later life.^5^ Birth weight is determined, in part, by intrauterine nutrition and shows a positive association with adult body mass index (BMI)^8^, ^9^ and a negative or U‐shaped association with abdominal obesity.^7^, ^10^ For obesity‐related diseases, a significant association has been observed between low birth weight and elevated risk of type 2 diabetes (T2DM)^11^, ^12^, ^13^, ^14^ and hypertension.^14^, ^15^, ^16^ It has been suggested that malnutrition in early life and overnutrition in later life may be the main drivers for the sharp increase in the prevalence of non‐communicable diseases observed in developing countries.^17^, ^18^

China has been experiencing an extremely rapid nutritional transition and has witnessed a sharp rise in the prevalence of obesity, T2DM, and hypertension over past half century, particularly during the past two decades.^19^, ^20^, ^21^, ^22^ Several studies based on survey data in China have shown that early life exposure to the 1959–1961 Chinese famine had long‐term adverse health consequences, including increased body mass index (BMI)^23^, ^24^ and elevated risk of T2DM^5^ and hypertension.^6^, ^23^, ^25^, ^26^ Due to the lack of information on birth weight at an individual level, direct evidence from this population is limited. So far, very few studies have investigated the association between birth weight and subsequent obesity,^7^, ^27^ whereas evidence is accumulating regarding an increased subsequent risk of T2DM^7^, ^27^, ^28^, ^29^ and hypertension^7^, ^27^ related to low birth weight.

In this study we used the data from two large prospective cohort studies conducted in China, the Shanghai Women's Health Study (SWHS) and the Shanghai Men's Health Study (SMHS), to evaluate associations of birth weight with multiple anthropometric measurements and estimate the relationship between birth weight and subsequent risk of T2DM and hypertension. The results may help us understand the causes and challenges of obesity, diabetes, and hypertension in China, and develop preventative strategies for the diseases as high priority for population health in low‐ and middle‐income countries.

## Methods

### Study population

The SWHS and the SMHS are both population‐based prospective cohort studies conducted in Shanghai, China. Details of the study methodologies have been reported previously.^30^, ^31^ Briefly, women aged 40–70 years and men aged 40–74 years living in eight study communities were approached to participate in the studies. During the period 1996–2000, 74 942 women were recruited to the SWHS (participation rate 92.7%), whereas during the period 2002–2006, 61 480 men were recruited to the SMHS (participation rate 74.1%).

### Baseline survey

At baseline, each participant completed an in‐person interview that collected information on demographics, birth weight, whether participants were breastfed as infants, lifestyle habits, dietary intake, physical activity habits, occupational history, and history of chronic diseases. The study protocols were approved by the relevant institutional review boards of all institutes involved, and written informed consent was obtained from all participants.

### Anthropometric measurements

At baseline, blood pressure, standing height (cm), body weight (kg), and waist and hip circumference (cm) were measured by trained health professionals according to standard protocols. Blood pressure was measured on the right arm using a standard mercury sphygmomanometer after participants had rested for at least 5 min. The first and fifth Korotkoff sounds were recorded. Standing height was measured to the nearest 0.1 cm without shoes. Body weight was measured to the nearest 0.1 kg using a digital scale that had been calibrated every 6 months. Waist circumference (WC) was measured 2.5 cm above the umbilicus and hip circumference was measured at the level of maximum protrusion of the gluteal muscles. Each measurement was taken twice with a tolerance of 1 mmHg for blood pressure, 1 cm for height and circumferences, and 1 kg for weight. If the difference between two measurements was greater than the tolerance, a third measurement was taken. The mean value of the two closest measurements was used in the present analysis. Body mass index was calculated as weight (kg) divided by height squared (m^2^). The waist:  hip ratio (WHR) and waist:  height ratio (WHtR), which have been shown to be better anthropometric indicators for adult cardiometabolic risk than BMI,^32^ were calculated by dividing WC by hip circumference (for WHR) and by standing height (for WHtR).

### Identification of prevalent T2DM and hypertension at baseline and incident cases since baseline and birth

Prevalent T2DM and hypertension at baseline were identified by asking participants whether they had ever been diagnosed with T2DM or hypertension by a physician and asking whether participants currently used hypoglycemic or antihypertensive medications. Participants who answered “Yes” to a T2DM‐related question were considered to have T2DM, and those who answered “Yes” to a hypertension‐related question were considered to have hypertension. In addition, participants with two abnormal blood pressure measurements at baseline (systolic blood pressure ≥ 140 mmHg or diastolic blood pressure ≥ 90 mmHg) were considered to have prevalent hypertension.

Cohort members were followed‐up for incident T2DM and hypertension, with blood pressure measurements taking place every 2–3 years after baseline. The follow‐up surveys for both cohort studies were organized by local health authorities. Therefore, home visits could be performed by trained retired nurses, which guaranteed high response rates of cohort members. For the SWHS, the response rates for the first (2000–2002), second (2002–2004), third (2004–2007) and fourth (2008–2011) in‐person follow‐up surveys were 99.8%, 98.7%, 96.7%, and 92.0%, respectively. For the SMHS, the response rates for the first (2004–08) and second follow‐up surveys (2008–2012) were 97.7% and 91.9%, respectively.

Both prevalent cases at baseline and incident cases since baseline were regarded as incident cases since birth. The vital status of participants was updated by annual record linkages to the Shanghai Vital Statistics Registry and the Shanghai Resident Registry.

### Statistical analysis

In all, 13 569 women and 11 515 men provided information on birth weight at the baseline survey and were included in the analyses. Participants were categorized into four groups according to birth weight (<2500, 2500–3499, 3500–3999 and ≥ 4000 g), with the 2500–3499 g group used as the reference category. Entry time was considered as time since birth or age at enrollment, which were used to calculate incidence since birth and since baseline, respectively. Exit time was defined as age at death, age at diagnosis of T2DM or hypertension, or last follow‐up contact date, whichever came first. We did not observe significant heterogeneity between men and women in associations between birth weight and subsequent risk of metabolic disorders; thus, we combined the two databases in the analysis.

The potential curvilinear relationships of birth weight with risk of adult obesity, T2DM, and hypertension were evaluated using restricted cubic splines functions (RCS)^33^ using the 5th, 25th, 75th, and 95th percentiles as fixed knots and the 50th percentile as the reference. Age was included as a spline variable to minimize residual confounding. Logistic regression models (odds ratios [ORs] and 95% confidence intervals [CIs]) were used to estimate associations of birth weight with the prevalence of obesity, T2DM, and hypertension, whereas Cox proportional hazard models (hazard ratios [HRs] and 95% CIs) were used to estimate associations of birth weight with the incidence of the diseases since birth and since baseline. Potential confounders included in the models were age (as a continuous variable), sex (male/female), education (no formal education or elementary school, middle school, high school, and college or above, as dummy variables), per capita income (<5000, 5000–10 000, and > 10 000 RMB for women; <12 000, 12 000–24 000 and > 24 000 RMB for men, as dummy variables), cigarette smoking (never/ever), alcohol consumption (never/ever), regular exercise (never/ever), and having been breastfed (never/ever), which were collected at baseline.

All analyses were performed by using SAS version 9.1 (SAS Institute, Cary, NC, USA), and all tests of statistical significance were based on two‐sided probabilities.

## Results

The prevalence of low birth weight (<2500 g) was 5.3% in men and 6.9% in women (Table 1). Men with higher birth weight, compared with those with lower birth weight, were older, more educated, more likely to use tobacco and alcohol, and were more likely to have been breastfed as infants, whereas women with higher birth weight did not differ in average age, cigarette smoking or alcohol consumption habits, but were more likely to have been breastfed.

**Table 1:** Baseline characteristics of participants according to birth weight, the Shanghai Women's Health Study and the Shanghai Men's Health Study

Characteristics	Birth weight (g)				*P*_trend_
	<2500	2500–3499	3500–3999	≥ 4000	
Men (SMHS)					
No. participants (%)	610 (5.3)	5818 (50.5)	2860 (24.8)	2227 (19.3)	–
Age at enrollment (years)	47.7 ±6.3	49.2 ±7.4	50.4 ±7.7	50.1 ±6.9	<0.0001
High educational achievement	22.8 [5.5]	27.1 [6.0]	24.5 [5.7]	23.4 [5.5]	0.0005
High income per capita*	9.6 [0.8]	11.8 [1.0]	13.0 [1.1]	11.1 [0.9]	0.05
Breastfed	83.3 [2.7]	92.6 [1.3]	95.4 [0.8]	95.6 [0.8]	<0.0001
Regularly smoked cigarettes	74.7 [8.3]	74.1 [8.4]	76.4 [8.0]	79.2 [7.4]	<0.0001
Regularly consumed alcohol	31.5 [2.3]	32.6 [2.3]	34.0 [2.4]	37.0 [2.5]	0.002
Regular leisure‐time activity	27.9 [11.9]	28.1 [12.0]	28.4 [12.0]	27.9 [11.9]	0.98
Women (SWHS)					
No. participants (%)	940 (6.9)	9320 (68.7)	1690 (12.5)	1619 (11.9)	–
Age at enrollment (years)	46.6 ± 6.5	48.5 ± 7.6	48.4 ±7.6	46.5 ±6.1	0.77
High educational achievement	15.5 [2.0]	18.3 [2.3]	17.7 [2.2]	17.7 [2.2]	0.20
High income per capita*	37.3 [4.3]	41.8 [4.5]	45.2 [4.6]	41.3 [4.5]	0.0011
Breastfed	78.0 [2.7]	91.2 [1.2]	94.2 [0.8]	94.2 [0.8]	<0.0001
Regularly smoked cigarettes	2.0 [1.2]	1.8 [1.0]	1.9 [1.1]	2.5 [1.4]	0.35
Regularly consumed alcohol	2.2 [0.04]	2.4 [0.05]	3.1 [0.06]	2.8 [0.05]	0.32
Regular leisure‐time activity	28.9 [11.6]	29.1 [11.6]	30.4 [11.8]	32.1 [12.0]	0.08

Data presented as n (%), as the mean ± SD, or as percentage [SD].

* High income per capita was defined as >24 000 RMB per capita per year for the Shanghai Men's Health Study (SMHS) and > 10 000 RMB for the Shanghai Women's Health Study (SWHS).

As indicated in Table 2, after adjusting for age, the average levels of BMI, WC, and WHtR increased as birth weight increased in both men and women (*P*_trend_ < 0.0001). However, the age‐adjusted mean WHR increased as birth weight increased, but only among men. The prevalence of T2DM and hypertension at baseline showed a U‐shaped relationship with birth weight.

**Table 2:** Baseline body size and prevalence of type 2 diabetes and hypertension according to birth weight, the Shanghai Women's Health Study and Shanghai Men's Health Study

	Birth weight (g)				*P*_trend_
	<2500	2500–3499	3500–3999	≥4000	
Men (SMHS)					
BMI at baseline (kg/m^2^)	23.20 (22.95, 23.45)	23.42 (23.33, 23.50)	24.08 (23.97, 24.20)	24.41 (24.28, 24.54)	<0.0001
WC at baseline (cm)	83.4 (82.7, 84.0)	84.2 (83.9, 84.4)	86.1 (85.7, 86.4)	87.1 (86.8, 87.5)	<0.0001
WHR at baseline	0.894 (0.889, 0.898)	0.895 (0.893, 0.896)	0.900 (0.898, 0.902)	0.905 (0.902, 0.907)	<0.0001
WHtR at baseline	0.494 (0.490, 0.498)	0.493 (0.492, 0.495)	0.500 (0.498, 0.502)	0.504 (0.502, 0.506)	<0.0001
Prevalence of T2DM	5.2 [3.4]	4.4 [3.0]	3.8 [2.6]	4.3 [2.9]	0.39
Prevalence of hypertension	25.4 [10.6]	22.1 [9.9]	21.9 [9.9]	24.1 [10.4]	0.05
Women (SWHS)					
BMI at baseline (kg/m^2^)	23.14 (22.93, 23.35)	23.35 (23.29, 23.42)	24.36 (24.21, 24.51)	24.36 (24.21, 24.52)	<0.0001
WC at baseline (cm)	75.40 (74.88, 75.91)	76.23 (76.06, 76.39)	78.50 (78.11, 78.88)	78.01 (77.62, 78.40)	<0.0001
WHR at baseline	0.804 (0.801, 0.808)	0.806 (0.805, 0.807)	0.809 (0.807, 0.811)	0.806 (0.803, 0.808)	0.19
WHtR at baseline	0.480 (0.477, 0.483)	0.481 (0.480, 0.482)	0.490 (0.488, 0.493)	0.487 (0.484, 0.489)	<0.0001
Prevalence of T2DM	4.0 [3.6]	2.5 [2.3]	1.9 [1.8]	3.2 [2.9]	0.0087
Prevalence of hypertension	22.0 [12.0]	18.2 [10.8]	18.6 [11.0]	17.3 [10.5]	0.02

Data for body measurements presented as the mean with 95% confidence limits in parentheses presented, whereas data regarding the prevalence of diseases presented as percentage [SD]. All data were adjusted for age as a continuous variable.

BMI, body mass index; SMHS, Shanghai Men's Health Study; SWHS, Shanghai Women's Health Study; T2DM, type 2 diabetes mellitus; WC, waist circumference; WHR, waist:  hip ratio; WHtR, waist:  height ratio.

**Figure 1:**
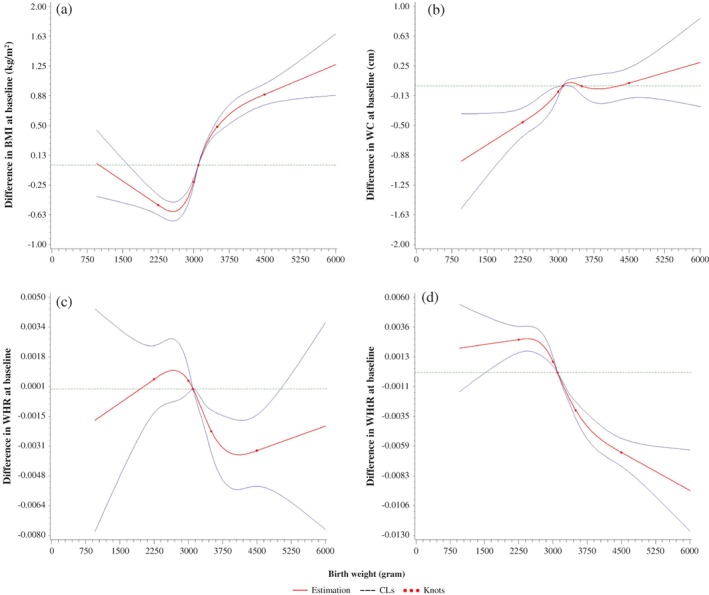
Non‐linear associations of birth weight and body size with a birth weight of 3100 g as the reference value using restricted cubic spline with 5 knots, adjusted for age, sex, education, income, smoking, alcohol consumption, having been breastfed and body mass index (BMI; for waist circumference [WC], waist: hip ratio [WHR] and waist: height ratio [WHtR]), from the Shanghai Women's Health Study and the Shanghai Men's Health Study. The solid lines are the estimates of differences in BMI, WC, WHR, and WHtR for any birth weight relative to a birth weight of 3100 g, and the dashed lines are confidence limits (CL). The horizontal dashed lines indicate the reference line where the difference was zero.

Figure 1 shows a significant non‐linear relationship between birth weight and obesity in adulthood. Body mass index at baseline decreased as birth weight increased up until approximately 2750 g, at which point BMI increased along with increasing birth weight, with a *P*‐value for overall significance of <0.0001, a *P*‐value for linearity of 0.0043 and a *P*‐value for non‐linearity of <0.0001. After adjusting for BMI, WC increased as birth weight increased (*P*‐value for overall significance <0.0001; *P*‐value for linearity = 0.0627; *P*‐value for non‐linearity = 0.0069), whereas WHR (*P*‐value for overall significance <0.0001; *P*‐value for linearity = 0.4045; *P*‐value for non‐linearity = 0.0199) and WHtR (*P*‐value for overall significance <0.0001; *P*‐value for linearity = 0.66; *P*‐value for non‐linearity <0.0001) increased until birth weight reached approximately 2750 g, after which WHR and WHtR decreased as birth weight increased. In addition, WHR increased further as birth weight increased past 4000 g.

Table 3 lists multivariable‐adjusted ORs and 95% CIs for baseline obesity according to birth weight. Compared with the median birth weight (3100 g), adjusted ORs for overall obesity at baseline associated with the 10th, 30th, 70th, and 90th percentiles of birth weight were 0.73 (95% CI 0.67–0.79), 0.89 (95% CI, 0.86–0.92), 1.32 (95% CI, 1.25–1.39), and 1.53 (95% CI, 1.40–1.67), respectively. Conversely, the associations of birth weight with central obesity differed greatly by the measures used. A significant association was observed for central obesity defined by WHR and WHtR (*P*‐value for overall significance <0.05), but not for that defined by WC (*P*‐value for overall significance = 0.20). Moreover, the association was linear for central obesity defined by WC (*P*‐value for non‐linearity = 0.1381) and WHR (*P*‐value for non‐linearity = 0.7731), but non‐linear for that defined by WHtR (*P*‐value for non‐linearity = 0.0068).

**Table 3:** Multivariable‐adjusted odds ratios and 95% confidence intervals for obesity according to birth weight, the Shanghai Men's Health Study and the Shanghai Women's Health Study

Birth weight (g)	Percentile	OR (95% CI)			
		For overall obesity*	For central obesity		
			By WC†	By WHR‡	By WHtR§
2500	10th	0.73 (0.67–0.79)	0.88 (0.78–0.98)	1.05 (0.96–1.14)	1.17 (1.04–1.32)
2750	20th	0.75 (0.69–0.81)	0.91 (0.82–1.01)	1.03 (0.96–1.12)	1.14 (1.02–1.27)
3000	30th	0.89 (0.86–0.92)	0.98 (0.93–1.01)	1.01 (0.98–1.04)	1.05 (1.01–1.10)
3000	40th	0.89 (0.86–0.92)	0.98 (0.93–1.01)	1.01 (0.98–1.04)	1.05 (1.01–1.10)
3100	50th	1.00 (Reference)	1.00 (Reference)	1.00 (Reference)	1.00 (Reference)
3250	60th	1.15 (1.12–1.17)	1.01 (0.98–1.04)	0.98 (0.96–1.00)	0.92 (0.89–0.95)
3500	70th	1.32 (1.25–1.39)	0.96 (0.89–1.04)	0.94 (0.89–1.00)	0.80 (0.74–0.86)
3650	80th	1.39 (1.29–1.49)	0.94 (0.85–1.04)	0.92 (0.86–1.00)	0.75 (0.67–0.83)
4000	90th	1.53 (1.40–1.67)	0.91 (0.81–1.03)	0.89 (0.81–0.98)	0.67 (0.59–0.77)
*P*‐values					
For overall significance		<0.0001	0.1963	0.0018	<0.0001
For linearity		0.0112	0.6918	0.8160	0.9337
For non‐linearity		<0.0001	0.1381	0.7731	0.0068

Multivariable‐adjusted odds ratios (ORs) were estimated using a logistic regression model with restricted cubic spline functions.

All ORs are adjusted for age, sex, education, per capita income, smoking, alcohol consumption, regular exercise, and having been breastfed; ORs for central obesity are additionally adjusted for body mass index (BMI) as a continuous variable.

CI, confidence interval.

* Defined as BMI ≥25 kg/m^2^.

† Referring to waist circumference (WC) ≥80 cm for women and ≥ 85 cm for men.

‡ Referring to a waist: hip ratio (WHR) >0.80 for women and > 0.90 for men.

§ Referring to waist: height ratio (WHtR) ≥0.50 for men and women.

**Figure 2:**
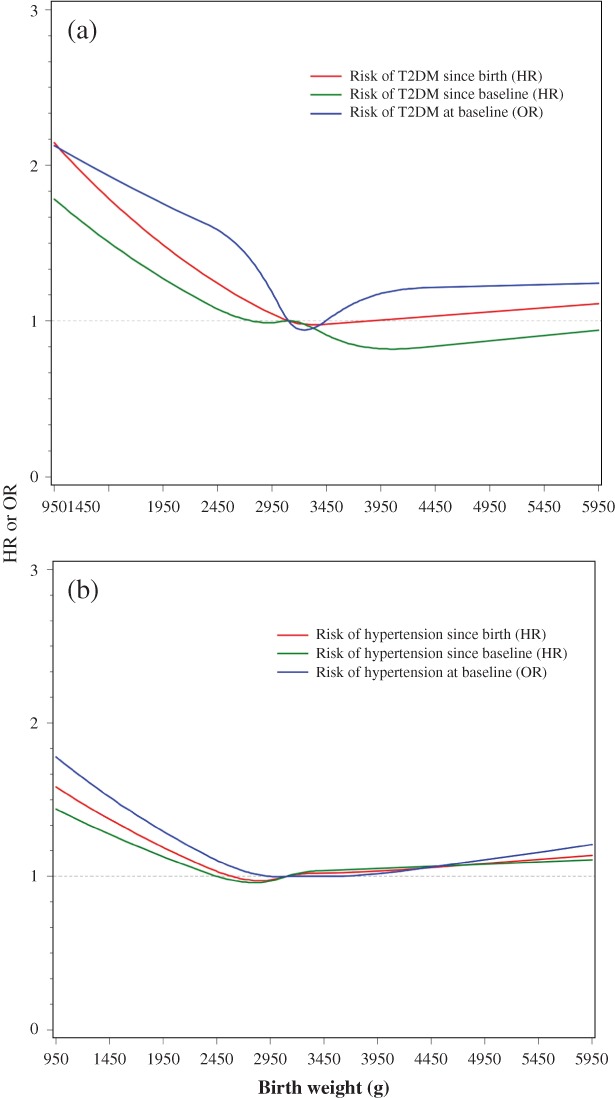
Smoothed plot for multivariable‐adjusted odds ratios (OR) or hazard ratios (HRs) for risk of (a) type 2 diabetes mellitus (T2DM) and (b) hypertension according to birth weight. The ORs were estimated by using a restricted cubic‐spline logistic with five knots placed at the 5th, 25th, 50th, 75th, and 95th percentiles of birth weight, whereas the HRs were estimated by using a restricted cubic‐spline proportional hazards model. The median value of birth weight (3100 g) was treated as the reference point. All ORs and HRs were adjusted for age, sex, education, income, smoking, alcohol consumption, and having been breastfed, from the Shanghai Women's Health Study and the Shanghai Men's Health Study.

As indicated in Fig. 2, the risk for T2DM and hypertension decreased as birth weight increased up to approximately 4000 g; further increases in birth weight did not appear to convey additional benefits (*P*‐value for non‐linearity <0.05 for the prevalence and incidence of T2DM and hypertension since birth, but *P* > 0.05 for incidence since baseline).

We further evaluated the associations of birth weight groups with the risk for T2DM and hypertension, and the results are presented in Table 4. Compared with normal birth weight (2500–3499 g), participants with low birth weight (<2500 g) were at higher risk for T2DM and hypertension, whereas high birth weight (≥3500 g) was associated with a lower risk of both diseases.

**Table 4:** Associations of birth weight with the risk of type 2 diabetes mellitus and hypertension, the Shanghai Men's Health Study and the Shanghai Women's Health Study

Diseases	Birth weight (g)				*P*_trend_
	<2500	2500–3499	3500–3999	≥4000	
Type 2 diabetes					
Prevalence at baseline (%)	3.74	3.23	3.27	3.62	
No. cases/non‐cases	58/1492	489/14649	149/4401	139/3707	
OR (95% CI)	1.40 (1.39–1.41)	1.00 (Reference)	0.79 (0.79–0.79)	1.04 (1.03–1.04)	<0.0001
Incidence since baseline					
No. cases	73	674	169	111	
Person‐years	12 255	122 756	31 558	27 628	
Incidence rate (1/1000)	5.96	5.49	5.36	4.02	
HR (95% CI)	1.17 (0.92–1.49)	1.00 (Reference)	0.95 (0.80–1.13)	0.76 (0.62–0.94)	0.0033
Incidence since birth					
No. cases	131	1163	318	250	
Person‐years	85 152	862 634	258 221	214 729	
Incidence rate (1/1000)	1.54	1.35	1.23	1.16	
HR (95% CI)	1.29 (1.07–1.54)	1.00 (Reference)	0.89 (0.78–1.01)	0.90 (0.78–1.03)	0.0019
Hypertension					
Prevalence at baseline (%)	20.84	19.86	21.54	20.55	
Hypertension/no hypertension	323/1227	3006/12132	980/3570	790/3055	
OR (95% CI)	1.27 (1.11–1.45)	1.00 (Reference)	0.99 (0.91–1.08)	1.04 (0.95–1.14)	0.36
Incidence since baseline					
No. cases	345	3250	1024	852	
Person‐years	10 170	104 199	26 553	23 771	
Incidence rate (1/1000)	33.92	31.19	38.56	35.84	
HR (95% CI)	1.13 (1.01–1.27)	1.00 (Reference)	1.05 (0.98–1.13)	1.06 (0.98–1.14)	0.54
Incidence since birth					
No. cases	668	6256	2004	1642	
Person‐years	83 552	847 575	254 484	211 944	
Incidence rate (1/1000)	8.00	7.38	7.87	7.75	
HR (95% CI)	1.20 (1.11–1.30)	1.00 (Reference)	1.02 (0.97–1.07)	1.04 (0.99–1.10)	0.67

Odds ratios (ORs) and hazard ratios (HRs) are adjusted for age, sex, education, smoking, drinking, regular exercise, and having been breastfed.

CI, confidence interval.

## Discussion

Using data from two prospective cohort studies of Chinese women and men, namely the SWHS and SMHS, we found non‐linear associations of birth weight with adult body size and risk for T2DM and hypertension. Low birth weight was generally associated with lower BMI, smaller WC, larger WHR and WHtR, and a higher risk for T2DM and hypertension. These findings are in agreement with most previous studies and indicate an important role of intrauterine nutrition in the etiology of subsequent metabolic disorders.

Low birth weight has been consistently associated with lower BMI but a higher risk of central obesity and metabolic syndrome in adults.^34^, ^35^ The Thrifty Phenotype Hypothesis was proposed by Hales and Barker^36^ to explain this pattern of associations. This hypothesis postulates that under conditions of suboptimal in utero nutrition, the fetus must adapt to its environment to ensure brain growth at the expense of other organs, such as the pancreas, heart, kidney, and skeletal muscle.^36^ During the in utero period, metabolic programming that promotes nutrient storage occurs in order to provide a survival advantage under conditions of poor postnatal nutrition. However, these adaptations can lead to the subsequent development of metabolic diseases, particularly under conditions of adequate postnatal nutrition or overnutrition.^37^

In the present study we found a J‐shaped relationship between birth weight and BMI at baseline, when participants were aged 40–74 years. Our finding of the lower birth weight–lower BMI relationship is in agreement with most previous studies conducted in Western populations,^34^, ^35^, ^38^ but is not consistent with studies conducted in Chinese adults.^7^ Based on data from the 2002 Chinese Nutrition and Health Survey, Yang et al.^39^ found that compared with women born in 1964, those born during the worst famine years (1959, 1960, and 1961) had higher BMI and a higher prevalence of overweightedness as adults. In a small‐scale study conducted in Chinese adults with a mean (±SD) age of 46.2 ±9.9 years, a significantly higher BMI was observed among those with low birth weight.^7^ The reasons for the inconsistency are not clear. However, low birth weight was complicated by presence of other qualitative or quantitative variables of social determinants for the nutrition transition in middle‐income countries.^18^ It is possible that the main problems may be unhealthy lifestyles in adulthood, which was not considered in the present and previous studies but is especially relevant in middle‐income countries that are going through a rapid transition from traditional to Western life patterns, such as China or India, where the prevalence of Western diet, smoking, sedentary lifestyle, insecurity food safety, obesity, and diabetes has increased markedly while the birth of children with low birth weight is still quite prevalent. More studies focusing on the effect of birth weight are needed to confirm the relationship.

It has been suggested that adverse exposures in early life can “program” short stature and a predisposition to abdominal adiposity, as well as an insulin resistance and other cardiometabolic risk factors in adult life.^37^ Low birth weight has been associated with a higher risk of central obesity, measured as WHR, after adjusting for adult BMI,^34^, ^40^, ^41^ although results are not consistent.^38^ Low birth weight was also linked to larger WC^7^ and WHtR^42^ in Asian populations. In the present study, we found that associations of birth weight with central obesity differed according to the measure of obesity being considered. Low birth weight was associated with smaller WC but larger WHR and WHtR. A non‐linear analysis showed that WC increased as birth weight increased, whereas WHR and WHtR were higher at lower birth weights. These results indicate that Chinese men and women with low birth weight may have a relatively small body size (i.e. shorter stature and smaller waist and hip circumference). In this population, WHR and WHtR, which are considered superior to WC for predicting adverse health outcomes,^32^ may be better indicators for birth weight‐related central obesity.

Accumulating evidence suggests that poor growth in utero, and thus low birth weight, is associated with an increased risk of developing diseases such as T2DM and hypertension in later life.^43^ Among individuals exposed to the Dutch Famine of 1944–45 during gestation, per kilogram birth weight was related to a 4.14‐mmHg decrease in systolic blood pressure, a 2.09‐mmHg decrease in diastolic blood pressure, and a 33% decrease in the risk of hypertension.^44^ A similar association was observed among Chinese people with prenatal exposure to the worst years of the Chinese famine (1959–61).^25^, ^26^ In recent years, a meta‐analysis summarizing 27 original studies showed an inverse linear association between birth weight and later risk of hypertension.^45^ Evidence is also accumulating for an association between low birth weight and risk of T2DM.^11^, ^12^, ^13^ A 1991 study found that men with the lowest birth weight (<2.5 kg) were nearly seven‐fold more likely to be glucose intolerant or have T2DM than men with the highest birth weight (>4.3 kg).^1^ Among 7874 rural Chinese adults born between 1954 and 1964, fetal exposure to the most severe period of the Chinese famine appeared to increase the risk of hyperglycemia in adulthood, and the association was exacerbated by a nutritionally rich environment in later life.^5^ Our findings of an elevated risk of T2DM and hypertension are consistent with these previous studies and provide further evidence supporting the Thrifty Phenotype Hypothesis proposed by Hales and Barker.^36^

The present study found no heterogeneity between sexes for associations of birth weight with adult body size and related chronic diseases. This result is not consistent with a previous study in which only women exposed to famine conditions in utero or during infancy were observed to have a greater risk of developing hypertension.^26^ The reason for this inconsistency is unclear. It is of note that in the present study 23.4% (143/610) of men with low birth weight were born during famine years, whereas only 2.9% (27/940) of women were born during famine years. Our results add evidence supporting a public health objective to reduce low birth weight in both sexes.

The strengths of the present study include the relatively large sample size and directly measured height, weight, and circumferences. However, several limitations should be acknowledged. First, birth weight was based on self‐report. We included only approximately one‐third of cohort members who answered the question on birth weight. Thus, both recall bias and selection bias are of concern. Second, a diagnosis of T2DM was also self‐reported. It is estimated that approximately half of all people with T2DM in China remain undiagnosed.^20^ Therefore, the association between birth weight and risk of T2DM may be biased towards null. Moreover, a residual confounding effect cannot be excluded, possibly due to the wide ranges of some categorical variables or unadjusted confounders, like adulthood lifestyle factors, social determinants, etc. Finally, the present study was conducted in urban Shanghai, the most economically developed city in China. The results in this population may not be applicable to other populations.

In conclusion, in the present study we found that low birth weight was associated with subsequent obesity and the prevalence and risk of T2DM and hypertension in Chinese men and women. The results suggest that nutrition in early life is of considerable importance to health in later life. Low birth weight should be considered as an important risk factor for obesity, diabetes, and hypertension in the general population and can be used to identify high‐risk individuals. Moreover, the government should pay more attention to maternal health to reduce the number of babies born with a low birth weight and thus to decrease the prevalence of obesity, diabetes, and hypertension in the general population in the long run.

## Disclosure

The authors declare no potential conflicts of interest.
